# Anticholesterolemic Activity of Three Vegetal Extracts (Artichoke, Caigua, and Fenugreek) and Their Unique Blend

**DOI:** 10.3389/fphar.2021.726199

**Published:** 2021-11-23

**Authors:** Jessica Frigerio, Erik Tedesco, Federico Benetti, Violetta Insolia, Giovanna Nicotra, Valerio Mezzasalma, Stefania Pagliari, Massimo Labra, Luca Campone

**Affiliations:** ^1^ FEM2-Ambiente, Milano, Italy; ^2^ Zooplantlab, Department of Biotechnology and Biosciences, University of Milano-Bicocca, Milano, Italy; ^3^ ECSIN-European Center for the Sustainable Impact of Nanotechnology, ECAMRICERT SRL, Padova, Italy; ^4^ EPO Srl, Milano, Italy

**Keywords:** anticholesterolemic activity, caigua, fenugreek, artichoke, botanicals, hepatic disease, choleretic activity

## Abstract

Hepatic-related diseases, in particular hyperlipidemia and hypercholesterolemia, are a thorn on the side of the national health institutes around the globe. Indeed, liver lipid and cholesterol dysregulation could lead to atherosclerotic plaque formation and cardiovascular diseases. Currently, statin administration and monacolin K consumption are the main therapies proposed to counter this alarming connection, but relevant side effects are known. To overcome this issue, safe nutraceutical formulations and/or vegetal extracts, endowed with anticholesterolemic activity, could be instrumental in hypercholesterolemia prevention and treatment. In the present work, the anticholesterolemic efficacy of three vegetal extracts used in traditional medicine (artichoke, caigua, and fenugreek), their unique blend (ACFB), and the monacolin K-containing red yeast extract (RYR), was investigated with an *in vitro* approach based on hepatic cell line HepG2. The impact on cholesterol of the three extracts, their blend, and RYR were investigated by determining hepatocyte total and free cholesterol and bile acids biosynthesis. According to our results, the anticholesterolemic activity of the vegetal extracts was confirmed, and a novel choleretic activity of caigua extract was evidenced. ACFB showed to be safer than RYR while showing a similar effect on total and free cholesterol and bile acids synthesis compared to it. The anticholesterolemic activity of the blend was obtained with lower vegetal extract concentrations compared with the single vegetal extract, potentially indicating an additive effect between the extracts. In conclusion, the vegetal extracts and their blend, ACFB, are safe and are endowed with anticholesterolemic activity, potentially providing complementary therapies to the statin-based ones for hyperlipidemia and hypercholesterolemia-related complications.

## Introduction

The liver is considered one of the most active organs in the human body (Ozougwu and Eyo, 2014). Indeed, the liver is a multifunctional organ, dealing with the regulation of many critical processes, such as bile secretion, metabolic detoxification, etc., and it is the mastermind behind nutrient metabolism and waste metabolites excretion. As such, it is not surprising that liver disease accounts for approximately 2 million deaths per year, equivalent to about 3.5% of all deaths worldwide (Rinella and Charlton, 2016; Asrani et al., 2019). In parallel with alcohol consumption, the progressive accumulation of fat in the liver (i.e., liver steatosis) is one of the main processes responsible for the onset and development of liver diseases. The liver is responsible for lipid homeostasis, regulating their absorption, distribution, and storage, β-oxidation, and lipogenesis (Ponziani et al., 2015; Pei et al., 2020). Among lipids in which homeostasis is regulated by the liver, cholesterol is undoubtedly one of the most important. Indeed, other than being a structural building block for all cell membranes (Liscum and Underwood, 1995; Simons and Ikonen, 2000; Grouleff et al., 2015), cholesterol is a cell signaling and neuronal conduction modulator (Orth and Bellosta, 2012; Sheng et al., 2012; Jin et al., 2019; Dai et al., 2021), and precursor to several relevant biomolecules such as steroid hormones, vitamin D, and bile acids (Russell, 2009). Cholesterol could be synthetized (*de novo* biosynthesis) by virtually all nucleated cells through the mevalonate pathway, a complex biochemical pathway mainly regulated by the activity of the HMG-CoA reductase (HMGCR), the primary rate-limiting enzyme in cholesterol biosynthesis. Beyond *de novo* cholesterol biosynthesis, responsible for two-thirds of the body cholesterol, cholesterol is also absorbed at the intestinal epithelium level, through a process known as exogenous pathway, involving several key proteins, such as the cholesterol transporter NPC1L1, the clathrin adaptor NUMB, and the adaptor protein LIMA1 (Altmann et al., 2004; Li et al., 2014; Afonso et al., 2018; Y-Y et al., 2018). Once within the cell, cholesterol is dynamically transported to the destined membranes for structural and functional needs (Morgan et al., 2016; Luo et al., 2019; Qi et al., 2020). However, when its absorption and/or *de novo* synthesis exceeds the cellular demand, cholesterol is either exported outside the cell by specific transporters, the ATP-binding cassette (ABC) transporters, or converted to cholesteryl esters by A:cholesterol acyltransferases (ACATs) and then stored in lipid droplets or secreted in the bloodstream via lipoproteins (Chang et al., 2006; Wang et al., 2017). However, while virtually all nucleated cells possess the molecular machinery to synthesize cholesterol (Arnold and Kwiterovich, 2003), only the liver, and in particular the hepatocytes, has the ability to eliminate cholesterol via bile secretion or its conversion into bile acids, with the latter now recognized as fundamental modulators of lipid, glucose, and energy metabolism through the activation of specific receptors (Russell, 2003; De Aguiar Vallim et al., 2013; Li and Chiang, 2015; Chiang and Ferrell, 2019). As such, the liver is the principal site for cholesterol homeostasis, mainly via biosynthesis, uptake through low-density lipoprotein receptors, lipoprotein release in the blood, storage by esterification and degradation, and conversion into bile acids (Weber et al., 2004). Liver failure in regulating cholesterol homeostasis, leading to hypercholesterolemia, is known to be a key point in cardiovascular disease development, such as coronary artery diseases (CAD), linked with the progressive accumulation of cholesterol at the atherosclerotic plaque level (Wider et al., 2016). Currently, statins (i.e. atorvastatin, fluvastatin, etc.) are the most widely prescribed drugs to lower plasma and hepatic cholesterol levels (Tiwari and Pathak, 2011) and, in response to the increased hypercholesterolemia population incidence, their use (and abuse) has grown exponentially. A clear example of this uncontrolled use is represented by monacolin K, the major representative of monacolins, natural statins present in *Monascus purpureus* Went (red yeast) extract (Nannoni et al., 2015). Despite their undeniable efficacy, some concerns were raised regarding statin- and monacolin-based therapy safety (Adhyaru and Jacobson, 2018). Regarding this topic, the European Food Safety Authority (EFSA) has recently published a scientific opinion on monacolin k safety, indicating that a dietary intake that does not give rise to concerns about harmful effects to health was not identified, for both the general population and vulnerable subgroups of the population (Younes et al., 2018). Regarding hypercholesterolemia problems, nutraceutical formulations have received considerable interest due to their nutritional, safety, and efficacy features. Indeed, the application of natural products, in particular, plant extracts, for cholesterol-related diseases, due mainly to fat-rich diet, is well rooted in the history of many populations. For example, the Mediterranean population reduced hypercholesterolemia-related conditions with the assumption of artichoke (*Cynara scolymus* L.) leaves or their extracts (Shimoda et al., 2003; Heidarian et al., 2011; Mohamed Abdel Magied et al., 2016), traditionally used as a diuretic and choleretic as well as for jaundice and liver insufficiency treatment (Wider et al., 2016). To the same end, the Brazilian population consumes caigua (*Cyclanthera pedata* Schrad.), an herbaceous vine better known as “maxixe do reino.” While its consumption is still very limited, recent studies have highlighted its potential use for hypercholesterolemia treatment, since it effectively lowers serum cholesterol level by regulating low- and high-density lipoproteins (LDL and HDL) (Gonzales et al., 1995; Montoro et al., 2001). In India and China (Asia), the side effects of a cholesterol-rich diet are traditionally dealt with the consumption of *Trigonella foenum-graecum* L. (fenugreek), an annual plant of the Fabaceae family. Fenugreek has been traditionally used for hypercholesterolemia, diabetes, coughs, congestion, bronchitis, fever, and high blood pressure (Basch et al., 2003; Vyas et al., 2008). The aim of the present work is to screen these novel vegetal extracts as single and uniquely blended in a novel nutraceutical formulation (artichoke caigua fenugreek blend; ACFB) for their anticholesterolemic activity at the hepatic level, as potential monacolin K substitute in hypercholesterolemia treatment. While *in vivo* studies remain the more reliable approach to investigate the effect of novel substances on liver metabolism, they are challenging due to the use of proxy measurements (e.g., the use of stable isotope tracers) and limited availability of liver biopsies (Steenbergen et al., 2013; Green et al., 2015). As such, for a preliminary screening of vegetal extracts and formulation efficacy and safety, an *in vitro* approach is usually preferred. For *in vitro* cellular studies, primary human hepatocytes are considered the best choice, but they present availability issues, interdonor variability, and a limited timeframe in which they remain differentiated (Steenbergen et al., 2013; Green et al., 2015). As a result, proliferating human hepatoma cell models, such as HepG2 cells, are the most widely used option. The human hepatoblastoma-derived cell line HepG2 is endowed with many functions attributed to a normal human hepatocyte. Indeed, this cell line secretes different plasma proteins (Knowles et al., 1980), including apolipoproteins, among which apoB-100 is the sole apoB species secreted by the human liver. Furthermore, HepG2 cells can synthesize and secrete lipoproteins, ranging from very low-density lipoprotein (VLDL) to high-density lipoprotein (HDL) (Rash et al., 1981; Zannis et al., 1981; Tam et al., 1985). Of the hepato-specific functions, these cells retain the ability to express the major regulatory enzymes of hepatic, plasma, and biliary cholesterol metabolism and are reported to synthesize and secrete bile acids including chenodeoxycholate and cholate (Everson and Polokoff, 1986). These activities respond in a manner consistent with what is known about human cholesterol metabolism *in vivo*, at least at a qualitative level. Moreover, HepG2 express receptors for insulin and transferrin (Ciechanover et al., 1983), estrogen (Tam et al., 1985), and LDL (Havekes et al., 1983; Dashti et al., 1984; Wu et al., 1984; Hoeg et al., 1985), and bind HDL (Hoeg et al., 1985), showing a close resemblance to primary hepatocyte receptor pool. Finally, HepG2 has a hepatocyte-like differentiated plasma membrane including a bile canalicular region, closely resembling primary hepatocyte morphology (Geuze et al., 1983).

The botanical identification of the three plant species was performed by DNA barcoding analysis, whereas the chemical characterization of the major constituent was performed by ultraperformance liquid chromatography coupled with quadrupole time of flight tandem mass spectrometry (UHPLC/QTof-MS). Moreover, in the present study, the anticholesterolemic and bile acid synthesis-promoting activity of ACFB is compared with two well-known anticholesterolemic substances, atorvastatin and *M. purpureus* extract.

## Materials and methods

### Materials

The Plant Genomic DNA Extract Mini Kit was purchased from Fisher Molecular Biology (Rome, Italy). The Qubit dsDNA HS Assay Kit was purchased from Invitrogen (Carlsbad, CA, USA). The PCR Mix Plus was purchased from A&A Biotechnology (Gdansk, Poland). HepG2 cell line (ATCC^®^ HB-8065™) was purchased from ATCC (Manassas, VA, USA). Dulbecco’s modified Eagle medium (DMEM) with GlutaMAX was purchased from Thermo Fisher Scientific (Waltham, MA, USA). Phosphate-buffered saline (PBS), penicillin–streptomycin mix, dimethyl sulfoxide (DMSO), and Cholesterol Quantitation Kit were purchased from Sigma-Aldrich (St. Louis, MO, USA). Fetal bovine serum (FBS) was purchased from Euroclone (Milan, Italy). CellTiter 96^®^ AQueous One Solution Cell Proliferation Assay (MTS) was purchased from Promega (Madison, WI, USA). The Total Bile Acid Assay Kit (Fluorometric) was purchased from Cell Biolabs San Diego, CA, USA). OriginLab software was purchase from OriginLab Corporation (Northampton, MA, USA).

### Methods

The determination of anticholesterolemic activity with an *in vitro* hepatic model, based on HepG2 cells, was performed on five samples, wherein each commercial name is reported in SI1: three vegetal extracts (*C. scolymus*, *C. pedata*, and *T. foenum-graecum*) and two formulations, *M. purpureus* extract (red yeast rice), 5% monacolin K (RYR), and ACFB. The latter formulation, known commercially as Omeolipid, is a polyextract of *C. scolymus*, *C. pedata*, and *T. foenum-graecum*, originating from the three vegetal simultaneous extraction (Supplementary Table S1).

#### Species identification by DNA barcoding

##### Sample collection and DNA extraction

Analyzed samples *Cyclanthera pedata*, *Trigonella foenum-graecum*, and *Cynara scolymus* were provided by EPO Srl. Among those provided as *Cyclanthera pedata*, one sample (DB690) came from some fresh fruits collected in Val Camonica (Italy) in November 2020. In order to analyze each batch representatively, five samples for each batch were collected. Fifty milligrams of dried plants were treated for DNA extraction by using the Plant Genomic DNA Extraction Mini Kit, following the manufacturer instructions. DNA for each sample was checked for concentration by using a Qubit 4.0 Fluorometer and Qubit dsDNA HS Assay Kit.

##### PCR condition, DNA sequencing, and species identification

The most suitable DNA barcode region for each species was chosen after an assessment of interspecific variability. Since *T. foenum-graecum* has high intraspecific variability, the gene *rbcL*, coding for the RuBisCO, was chosen as the most effective marker. For the species *C. pedata* and *C. scolymus*, the intergenic spacer *psbA-trnH* was chosen, due to its high power of discrimination. The primers used for DNA amplification are described in Supplementary Table S2. PCR amplification was carried out using PCR Mix Plus. The mix reaction is composed of 12.5 µl of PCR Mix Plus, 1 µl of each primer (10 µM), 7.5 µl of sterile water, and 3 µl of genomic DNA (30–50 ng). PCR cycles consisted of an initial denaturation step (7 min at 94°C), followed by 35 cycles of denaturation (45 s at 94°C), annealing (30 s at 50°C for rbcL and 53°C for psbA-trnH), and extension (1 min at 72°C), and a final extension step at 72°C for 7 min. Amplicon presence was assessed by electrophoresis on 1.5% agarose gel. Purified amplification products were bidirectionally sequenced by Eurofins Genomics. The 3′ and 5′ terminal portions of each sequence were clipped to generate consensus sequences for each sample. All sequences were manually edited, the primer was removed, and after pairwise alignment, the obtained sequences were identified by a standard comparison approach against a GenBank database with Basic Local Alignment Search Tool (BLAST) (https://blast.ncbi.nlm.nih.gov/). Each barcode sequence was taxonomically assigned to the plant species with the nearest matches (maximum identity >99% and query coverage of 100%) according to Galimberti and colleagues (Galimberti et al., 2016).

#### UHPLC-DAD-HRMS/MS profiles of ACFB

The identification of compounds in the extracts was carried out using using a Waters ACQUITY UPLC system coupled with a Waters Xevo G2-XS QTof Mass Spectrometer (Waters Corp., Milford, MA, USA). All analytes were separated on a Kinetex Biphenyl (100 mm × 2.1 mm, 2.6 µm). The mobile phases were both MS grade H_2_O (A) and CH_3_CN (B), both containing 0.1% formic acid (HCOOH), with gradient elution as follows: 0–2.0 min, 5–10% B; 2.0–17.0 min, 10–35% B; 17.0–18.0 min, 35%–95%, after each run of 5 min of wash (98% B), and 5 min of equilibration was performed before the next sample injection. Elution was performed at a flow rate of 400 µl min^−1^, and the injection volume was 10 μl. The column temperature was set to 30°C. UV spectra were acquired in the range of 210–400 nm, and two wavelengths, 280 and 330 nm, were employed for the detection of target analytes. The Xevo G2-XS QTof Mass Spectrometer equipped with an ESI source, was used in negative and positive ionization modes to acquire full-scan MS, and the spectra were recorded in the range of m/z 100–1,000. The source parameters were as follows: electrospray capillary voltage 2.5 kV, source temperature 150°C, and desolvation temperature 500°C. The cone and desolvation gas flows were 10 and 1,000 L/h, respectively. A scan time of 0.5 s was employed. The cone voltage was set to 60 V, and ramping collision energies ranged from 6 to 30 V to produce abundant product ions before detection at the Tof. The mass spectrometer was calibrated with 0.5 M sodium formate, and leucine-enkephalin (100 pg/μl) was used as LockMass (m/z 554.2615, 2 kV ionization voltage), which was infused simultaneously with the flow of column at 10 μl/min and acquired for 1 s each 10 s. The base peak chromatograms (BPI) were acquired at low (6) and high (30) energy from which the peak identification was performed. From the low-energy spectra, the molecular ion mass [M – H]– was obtained from which the elementary composition was calculated (mass error <5 ppm), while from the high-energy spectra, the fragmentation pattern was obtained, and the information was used for the identification. Phenolic compounds were characterized according to the corresponding spectral characteristics (UV and MS spectra [M – H]−), accurate mass, characteristic fragmentation, and consulted different databases (PubChem, ChemSpider, and KEGG). The MassLynx software (version 4.2) was used for instrument control, data acquisition, and data processing.

#### HepG2 cell culture

The human epithelial hepatocellular carcinoma HepG2 cells (passage 90–95) were maintained in HepG2 cell culture medium (HepG2 CCM) (DMEM + GlutaMAX medium supplemented with 10% FBS and 1% penicillin–Streptomycin mix). The cells were grown in a controlled atmosphere incubator (85% relative humidity, 5% CO_2_, and 37°C). HepG2 cells were seeded at 20,000 cell/cm^2^, and the medium changed every other day. Cells were subcultivated by trypsinization every 4 days, when 80%–90% confluent.

#### Solubilization of vegetal extracts, ACFB, and red yeast rice

The three vegetal extracts were resuspended in water up to a concentration of 750 mg/ml for *C. scolymus* and *C. pedate* extracts and 400 mg/ml for *T. foenum-graecum* extract. ACFB was easily resuspended in water to a concentration of 300 mg/ml, while RYR shown to be sparingly soluble in the same solvent. To improve the solubilization of monacolin K, RYR was resuspended in DMSO to a concentration of 200 mg/ml. This stock was further diluted in HepG2 culture medium to a concentration of 100 mg/ml, vortexed, centrifuged to pellet insoluble material, and finally, the resultant supernatant was collected. Monacolin K concentration in the supernatant was determined by HPLC (high-performance liquid chromatography). Since the concentration of extracted monacolin K was low (0.08 μg/ml), we tried to improve monacolin K solubilization with different solvents (i.e., ethanol and methanol), following the indications provided by the work of Singgih and colleagues (Singgih et al., 2014). Briefly, RYR was resuspended in methanol and ethanol, agitated with a shaker for 2 h at RT, and the supernatant was collected following centrifugation. At the end of the solubilization process, a spectrophotometric analysis of the obtained supernatants was performed, and the solubilization efficiency was evaluated by comparing their absorption at 240 nm (absorption peak for monacolin K) with that resulting from DMSO-based solubilization. Considering the different solvents in which the vegetal extracts of the ACFB and the RYR were resuspended, preliminary experiments were conducted to rule out any solvent-dependent effect on the considered endpoints (i.e., bile acids synthesis and cholesterol metabolism).

#### Cell viability assay on human hepatic cell line

To evaluate the impact of artichoke, caigua, and fenugreek extracts on HepG2 cells, and to determine their higher, non-toxic concentration, a dose–response curve experiment on HepG2 cells was performed. Briefly, HepG2 cells were seeded on 96-multiwell plates and left to adhere and propagate for 24 h. Following incubation, HepG2 cells were treated with increasing concentrations of caigua extract (from 0 to 75 mg/ml), fenugreek extract (from 0 to 40 mg/ml), and artichoke extract (from 0 to 75 mg/ml) for 48 h, corresponding to the duration of anticholesterolemic activity experiments. At the end of the incubation time, treated HepG2 cells were carefully washed with PBS, and their viability was determined by MTS assay, according to the instruction of the manufacturer. The same approach was applied to ACFB (from 0 to 10 mg/ml), RYR (from 0 to 2 mg/ml), and atorvastatin (positive control; from 0 to 9. 1 μg/ml), a statin medication used to treat abnormal lipid levels and to prevent cardiovascular disease in those at high risk (Lennernäs, 2003). Viability results were expressed as a percentage (%) compared with the negative control (HepG2 CCM-treated cells). Obtained results were fitted with OriginLab, and the EC50 (half-maximal effective concentration) value was calculated.

#### Anticholesterolemic activity evaluation

The specific anticholesterolemic activity at the hepatic level of the three vegetal extracts, ACFB, RYR, and atorvastatin was evaluated in a human hepatic cell line (HepG2). Briefly, HepG2 cells were seeded in six multiwell plates and were made to adhere and proliferate for 24 h. At the end of the incubation, HepG2 were exposed, for 48 h in controlled conditions, to the highest, non-toxic concentration of the three vegetal extracts, ACFB, and RYR, according to the dose–response results. For the positive control atorvastatin, a concentration comparable with that available in literature was applied (Han et al., 2017). At the end of the treatment, the hepatic cells were thoroughly washed with prewarmed PBS and processed for the determination of bile acids and the different forms of cholesterol.

##### 1.2.6.1 Hepatic cholesterol biosynthesis evaluation

The anticholesterolemic activity of ACFB and RYR was evaluated by determining the total, free, and esterified cholesterol fractions following treatment (Guo et al., 2017). The different fractions were assessed with a commercial kit, Cholesterol Quantitation Kit, following the instructions of the producer. Briefly, at the end of the treatment with the three vegetal extracts, ACFB, RYR, and atorvastatin, treated HepG2 were collected by trypsinization, pelleted by centrifugation, and extracted with chloroform:isopropanol:IGEPAL^®^ CA-630 (7:11:0.1 ratio) in a microhomogenizer. Then samples were centrifuged at 13,000 × *g* for 10 min to pellet insoluble material and the organic phase transferred to a new tube and air dried at 50°C to remove chloroform. To remove any residual organic solvent, samples were put under a vacuum for 30 min. Obtained dried lipids were resuspended in an appropriate buffer and sonicated until the mixture was homogenous. The different cholesterol fractions were measured with a coupled enzymatic reaction, which ensures the direct proportionality between the produced fluorescence (excitation wavelength 535 nm; emission wavelength 587 nm) and the concentration of the different cholesterol forms. Fluorescence readings were performed with a multiwell plate reader (Synergy4, Biotek). The results for total, free, and esterified cholesterol were normalized on sample protein concentration and expressed as a percentage (%) compared with the negative control (untreated hepatic cells).

##### Determination of bile acid production

The production of bile acids by the HepG2 cells, following treatment with vegetal extracts, ACFB, RYR, and atorvastatin, was evaluated with a fluorimetric assay (total bile acid assay) (Shao et al., 2016). Briefly, at the end of the treatment (48 h), cells were thoroughly washed with cold PBS before lysis, which was performed by sonication in cold PBS. Then lysates were centrifuged at 10,000 × *g* for 10 min at 4°C, and obtained supernatants were assayed for the presence of bile acids. Bile acid determination is based on the production of a fluorescent substrate (resorufin; excitation wavelength 560 nm and emission wavelength 590 nm) in their presence, due to the coupled activity of two enzymes. As for the cholesterol, obtained results were normalized on the protein concentration and expressed as a percentage (%) compared with the negative control (untreated hepatic cells).

#### Statistical analysis

Results were statistically analyzed by t-test, using OriginLab software (OriginLab Corporation, Northampton, MA, USA). Experiments were performed in triplicate, and results were presented as average ± standard deviation. For ACFB UHPLC-DAD-HRMS/MS profiles statistical analysis, analysis of variance (ANOVA) was used to compare the means, while Turkey’s test was used to assess the statistically significant differences among treatments. A *p*-value of ≤0.05 was considered significant.

## Results

### DNA barcoding results

DNA barcoding approach is a widely used molecular-based identification system based on the analysis of the variability within a standard region of the genome. An ideal DNA barcode requires high taxonomic coverage and high resolution (Hebert et al., 2003). As a general principle, a DNA barcode region should have a high interspecific and low intraspecific variability. Good DNA quality (i.e., A260/A230 and A260/A280 within the range 1.8–2.2) and extraction yield (i.e., 20–40 ng/μl) were obtained for all the analyzed samples. Each barcode sequence was taxonomically assigned by using BLASTn analysis to the plant species with the nearest matches (maximum identity >99% and query coverage of 100%). All the samples returned 100% maximum identity (with 100% query coverage). As shown in Supplementary Table S3, the results of DNA barcoding confirmed the declared species for all the samples, a fundamental step to obtain the suitable phytocomplexes for ACPB regarding the sample DB690. The results confirmed that the fruit known as “milione” or “miliun” is effectively *Cyclanthera pedata*, demonstrating, for the first time, to the best of our knowledge, that caigua is also grown in Val Camonica (Lombardy, Italy). This finding could positively impact the agriculture of this mountainous area, leading to a more sustainable supply chain for its use as food and in food supplements.

### UHPLC-ESI-HRMS untarget analysis of ACFB

**FIGURE 1:**
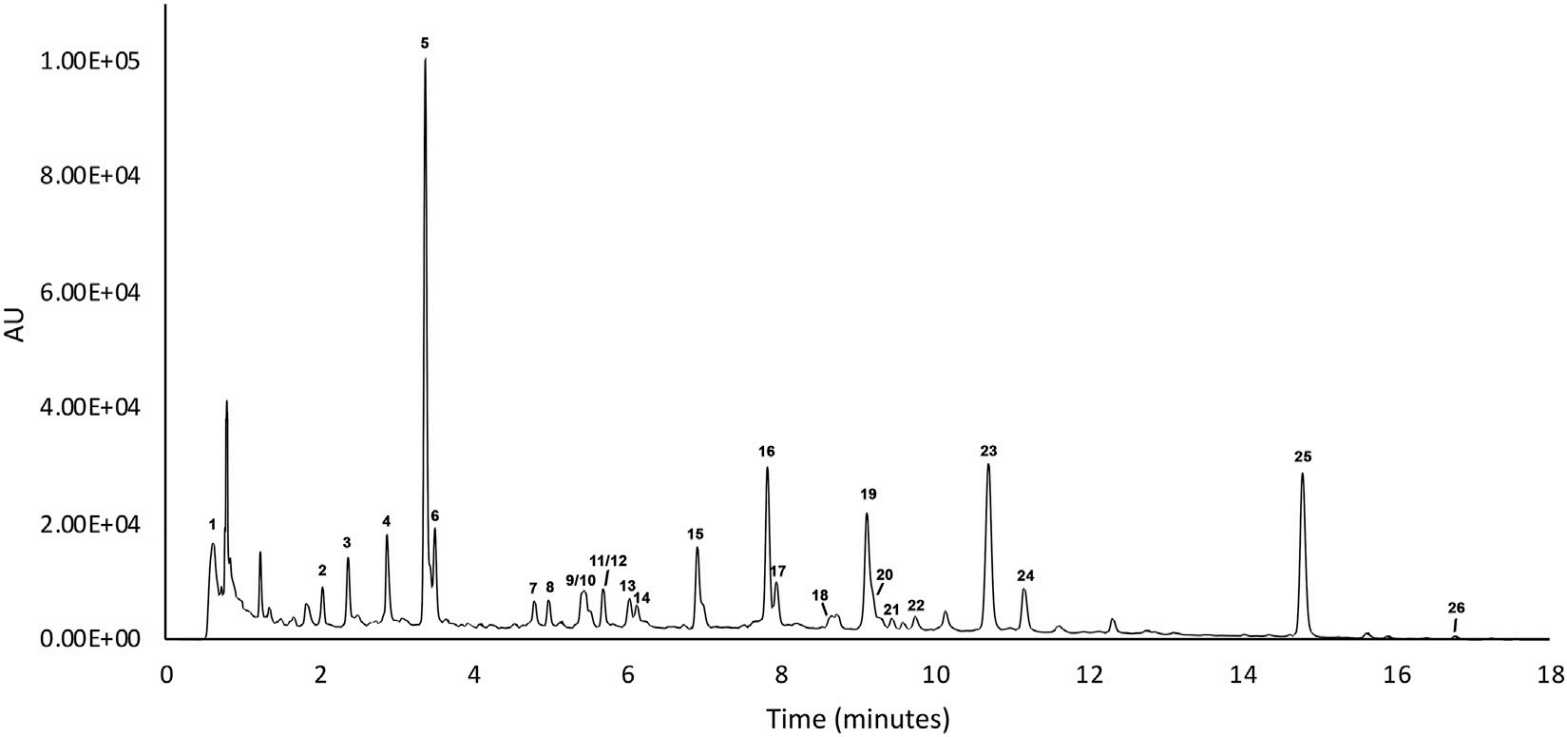
UV chromatograms of ACFB sample recorded at 280 nm.

In order to verify the content of active substances within the blended extract (ACFB), the UHPLC-ESI-HRMS analysis was carried out. Initially, to obtain a good chromatographic separation and improve ionization of compounds, mobile phase composition, columns, and elution gradient were optimized. UHPLC profiles were acquired with UV (280–330 nm) and by HRMS. The MS data both in positive and negative ionization modes were acquired to obtain a fully and complementary structural information, and each metabolite was fragmentated to allow a deep structural elucidation. The metabolite identification was carried out by using UV spectra, HRMS data (accurate mass, isotopic distribution, and MS/MS characteristic fragmentation pathway), and literature databases. Finally, the tentatively identified compounds were confirmed with the standard whenever available. UHPLC-UV chromatograms of ACFB at 280 nm are given in Figure 1 and in Table 1 reports the list of the 255 identified phytochemicals numbered according to elution order. The UHPLC-UV-HRMS/MS analysis allowed the identification of 266 metabolites belonging mainly to two different classes: flavone and quinic acid (Table 1). The flavones were detected in the extracts in both C and O glycosidic form. Ten flavone C-glucosides (7, 8, 10–15, 23–25) were detected, and the fragmentation pathway that was observed in their MS2 spectra confirmed the structure of the identified compounds (Farag et al., 2013; Celano et al., 2019). These flavone c-glucosides in their (−) MS2 spectra produced a characteristic fragmentation pathway, showing successive or simultaneous losses of the glycosylic group (60, 90, and 120 Da) corresponding to [M−H-pentose] and/or [M−H-hexose], respectively. Four flavone O-glucosides detected were putatively identified as glycoside derivatives of apigenin and luteolin, respectively, based on molecular formulae, product ions, literature data, and comparison with reference standards (16). Another class of detected compounds belong to the quinic acid compounds, in particular, mono- and di-caffeoylquinic acids. With regard to mono-caffeoylquinic acid, four compounds were detected (21, 3, 5, 64) showing the same [M−H]– at m/z 352.0880 in accordance with the molecular formula C16H17O9–. In their (−) MS2 spectra, the fragmentation ion at m/z 191 was produced, which represents a quinic acid, resulting from neutral loss of caffeic acid (Sanchez-Rabaneda et al., 2003; Moglia et al., 2008). On the basis of these data, these compounds have been assigned as mono-caffeoylquinic acid and its isomers. In addition, compounds 97, 187, 198, 221, with the same precursor ion at m/z 515.1194 and identical molecular formula C25H23O10– in their (−) MS2 spectra showed the typical fragmentation pathway of di-caffeoylquinic acid isomer (Sanchez-Rabaneda et al., 2003; Moglia et al., 2008). Finally, the latter identified compound, not visible in the chromatogram UV at 280 nm but clearly visible only in positive mode, is the alkaloid trigonelline (1). The alkaloid trigonelline show [M + H]+ at m/z 138.0551 in accordance with the molecular formula C_7_H_8_NO_2_^+^, and in the (+) MS2 spectra the fragmentation ion at m/z 92 and 94 was produced, in accordance with literature data (Oldoni et al., 2019).

**TABLE 1:** List of compounds deteced following UHPLC-ESI-HRMS untarget analysis of ACFB.

N°	Rt (min)	[M − H]−	[M + H]+	Formula	Δ ppm^a^	MS/MS^b^	Name	Class	Ref
1	0,61	/	138,0551	C7H8NO2	−2.9	92/94	Trigonelline	Alkaloid	Oldoni et al. (2019)
2	2.03	353.0880	355.1079	C16H17O9	2.0	191	Mono-caffeoylquinic acid isomer	Quinic acid	Abu-Reidah et al. (2013)
3	2.36	353.0880	355.1079	C16H17O9	2.0	191	Mono-caffeoylquinic acid isomer	Quinic acid	Abu-Reidah et al. (2013)
4	2.88	433.0511	435.0626	/	/	415/387/258/215/191/161	Unknown	/	/
5	3.36	353.0880	355.1079	C16H17O9	2.0	191	Mono-caffeoylquinic acid isomer	Quinic acid	Abu-Reidah et al. (2013)
6	3.49	353.0880	355.1079	C16H17O9	2.0	191	Mono-caffeoylquinic acid isomer	Quinic acid	Abu-Reidah et al. (2013)
7	4.78	593.1523	595.1665	C27H29O15	2.9	503/473/383/353	Apigenin 6.8-di C-hexoside (vicenin2) isomer	Flavone-C-glycoside	M.A. Farag et al. (2014)
8	4.97	593.1523	595.1665	C27H29O15	2.9	503/473/383/353	Apigenin 6.8-di C-hexoside (vicenin2) isomer	Flavone-C-glycoside	M.A. Farag et al. (2014)
9	5.36	515.1194	/	C25H23O12	0.8	353/179/173/191	Di-caffeoylqyuinic acid isomer	Quinic acid	Abu-Reidah et al. (2013)
10	5.4	563.1385	565.1635	C26H27O14	−2.8	503/473/443/383/353	Apigenin-6-C-hexoside-8-C-pentoside (vicenin 3) isomer	Flavone-C-glycoside	M.A. Farag et al. (2014)
11	5.68	447.0918	449.1126	C21H19O11	−2.0	357/327	Luteolin-6-C-glucoside (isoorietin)	Flavone-C-glycoside	M.A. Farag et al. (2014)
12	5.68	447.0918	449.1126	C21H19O11	−2.0	357/327	Luteolin-8-C-glucoside(orientin) isomer	Flavone-C-glycoside	M.A. Farag et al. (2014)/Orsini et al. (2019)
13	6.02	563.1385	565.1635	C26H27O14	−2.8	503/473/443/383/353	Apigenin-6-C-hexoside-8-C-pentoside (vicenin 3) isomer	Flavone-C-glycoside	M.A. Farag et al. (2014)
14	6.12	447.0918	449.1126	C21H19O11	−2.0	357/327	Luteolin-8-C-glucoside(orientin) isomer	Flavone-C-glycoside	M.A. Farag et al. (2014)/Orsini et al. (2019)
15	6.9	431.0998	433.1181	C21H19O10	4.6	311/341/283	Apigenin-6-C-glucoside (isovitexin)	Flavone-C-glycoside	Orsini et al. (2019)
16	7.82	447.0943	449.1126	C21H19O11	3.6	285	Luteolin-7-O-glucoside (cynaroside)	Flavone-O-glycoside	Abu-Reidah et al. (2013)
17	7.93	461.0706	463.0952	C21H17O12	−3.0	285	Luteolin-7-O-glucuronide	Flavone-O-glycoside	Abu-Reidah et al. (2013)
18	8.55	515.1194	/	C25H23O12	0.8	353/179/173/191	Di-caffeoylqyuinic acid isomer	Quinic acid	Abu-Reidah et al. (2013)
19	9.11	515.1194	/	C25H23O12	0.8	353/179/173/191	Di-caffeoylqyuinic acid isomer	Quinic acid	Abu-Reidah et al. (2013)
20	9.17	431.0976	433.1181	C21H19O10	−0.5	269	Apigenin-7-O-glucoside (cosmoside) I	Flavone-O-glycoside	Abu-Reidah et al. (2013)
21	9.44	445.0766	447.098	C21H17O11	−1.1	269	Apigenin-7-O-glucoronide I	Flavone-O-glycoside	Abu-Reidah et al. (2013)
22	9.74	515.1194	/	C25H23O12	0.8	353/179/173/191	Di-caffeoylqyuinic acid isomer	Quinic acid	Abu-Reidah et al. (2013)
23	10.69	399.1156	401.1355	C21H19O8	−1.3	325/295/267	Chrysin 6-C-fucopyranoside	Flavone-C-glycoside	Orsini et al. (2019)
24	11.16	415.1027	417.1251	C21H19O9	−0.5	325/295/267	Chrysin derivative	Flavone-C-glycoside	Orsini et al. (2019)
25	14.78	399.1156	401.1295	C21H19O8	−1.3	325/295/267	Chrysin 6-C-fucopyranoside	Flavone-C-glycoside	Orsini et al. (2019)
26	16.8	441.1200	443.1355	C23H21O9	3.2	381/337/307/295	Chrysin derivative	Flavone	Orsini et al. (2019)

### Cytotoxicity evaluation of the three vegetal extracts, ACFB, and red yeast rice

**FIGURE 2:**
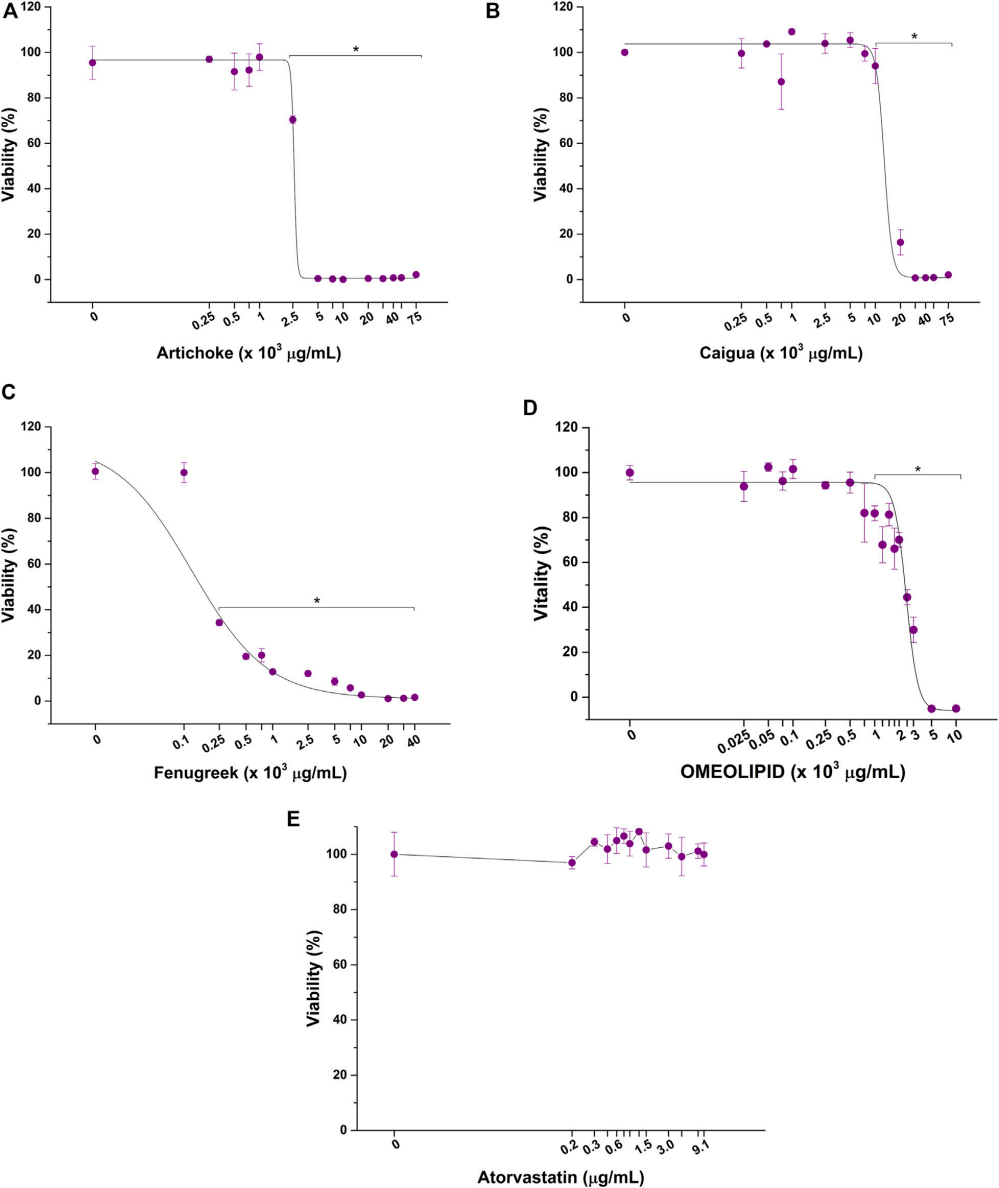
Cytotoxic effect of artichoke **(A)**, caigua **(B)**, fenugreek **(C)**, ACFB, **(D),** and atorvastatin **(E)** on hepatic *in vitro* model viability following 48 h of exposure. *p < 0.05.

Before exploring the potential anticholesterolemic activity of the three vegetal extracts, ACFB, atorvastatin, and RYR, their impact on HepG2 cell viability was evaluated by dose–response toxicological analysis, considering 48 as relevant exposure times. As shown in Figure 2, a significant reduction in HepG2 cell viability is observed following exposure to a concentration starting from 2,500 μg/ml for artichoke (Figure 2A), 10,000 μg/ml for caigua (Figure 2B), and 250 μg/ml for fenugreek (Figure 2C). As such, the artichoke extract is the more cytotoxic among those tested, as indicated by its lower EC50 (Table 2), while caigua showed to be the safest one. The vegetal extracts blend, ACFB, significantly lowered HepG2 viability starting from 1,000 μg/ml (about 20% viability reduction) (Figure 2D). However, a significant effect on hepatic cell morphology was highlighted even at lower concentrations (750 μg/ml) (Supplementary Figure S1). No adverse effect on HepG2 cell viability was observed for atorvastatin at all tested concentrations (Figure 2E).

**TABLE 2:** EC50 values of the vegetal extracts, their blend (ACFB), and RYR solubilized in different solvents (ethanol, methanol, and DMSO).

Treatment	EC50 (µg/ml)
Artichoke extract	2,763.9 ± 299.8
Caigua extract	12,790.9 ± 4,418.2
Fenugreek extract	139.5 ± 49.8
ACFB	2,435.7 ± 93.6
Red yeast rice(RYR) (ethanol)	424.0 ± 7.0
RYR (methanol)	238.0 ± 14.0
RYR [dimethyl sulfoxide (DMSO)]	>2,000
Atorvastatin	>9.1

Note. The results are reported as mean ± standard deviation.

**FIGURE 3:**
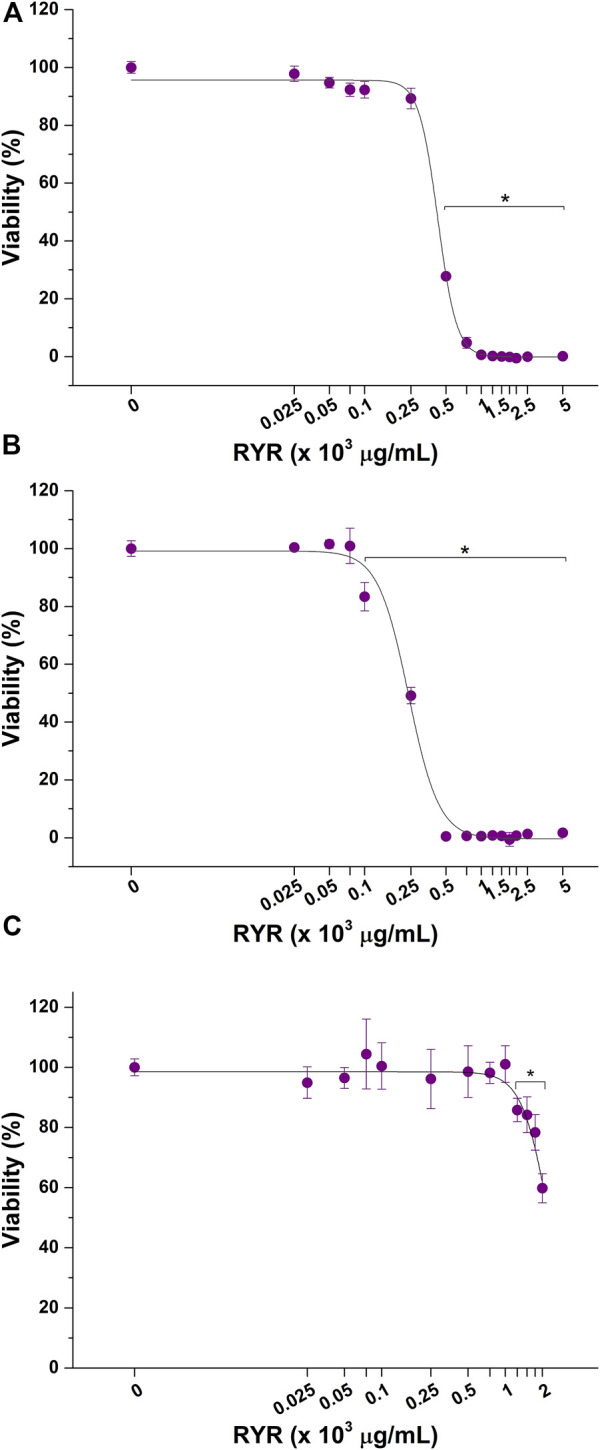
Cytotoxic effect of red yeast rice (RYR) solubilized in ethanol **(A)**, methanol **(B)**, and dimethyl sulfoxide (DMSO) **(C)** on hepatic *in vitro* model viability following 48 h of exposure. *p < 0.05.

The cytotoxicity of RYR solubilized in DMSO, ethanol, and methanol was also tested. As evidenced by dose–response curves reported in Figure 3, RYR solubilized with methanol significantly affected HepG2 cell viability at lower concentrations compared with RYR solubilized in ethanol (100 versus 500 μg/ml) (Figures 3A, B). No adverse effect on hepatic *in vitro* model viability was observed up to 1,000 μg/ml when RYR is solubilized in DMSO (Figure 3C). As confirmed by EC50 values reported in Table 2, RYR solubilized in DMSO is less cytotoxic, while RYR in methanol showed the highest toxicity (i.e., lowest EC50). As for ACFB, morphological alteration of HepG2 cells was observed down to 500 μg/ml for DMSO-solubilized RYR (data not shown). In the light of the obtained results, the anticholesterolemic activity assessment was performed at 1,000, 7,500, and 100 μg/ml for artichoke, caigua, and fenugreek extracts, respectively. To ensure a better comparison, ACFB and RYR were solubilized in DMSO and tested at the same concentration (250 μg/ml).

### Effect of vegetal extracts, ACFB, and red yeast rice on bile acid biosynthesis

**FIGURE 4:**
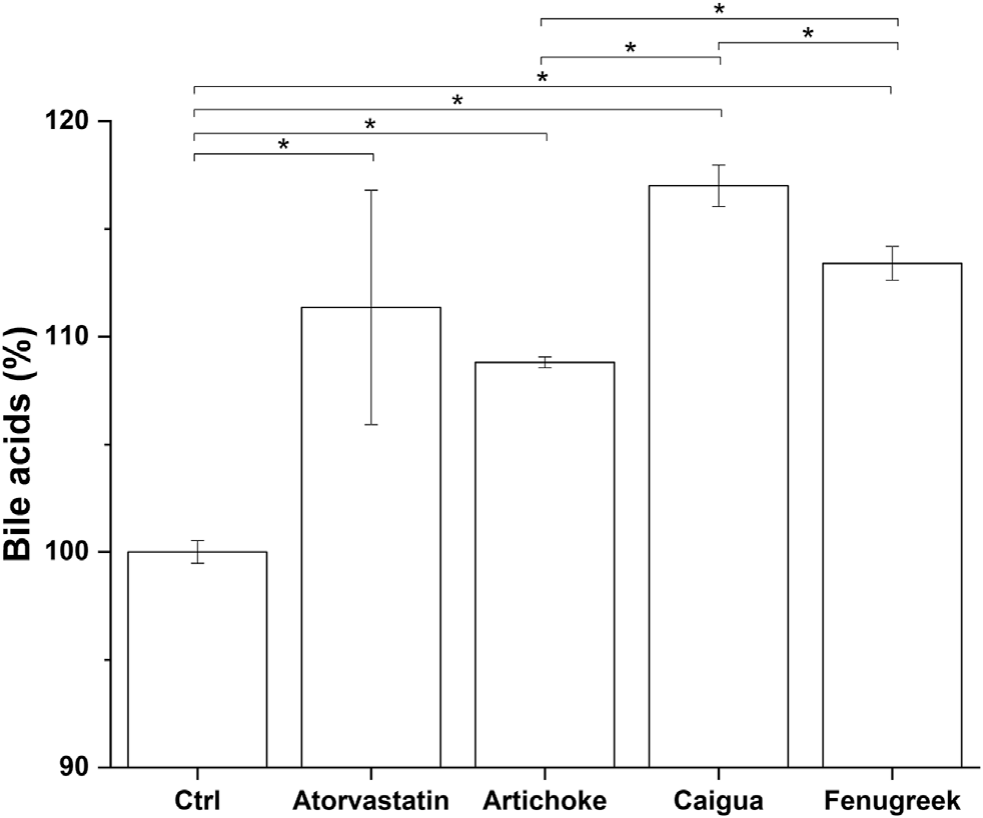
Bile acid production following treatment of hepatic *in vitro* model with atorvastatin (positive control) and the highest, no-toxic concentration of artichoke, caigua, and fenugreek extracts. Monacolin K concentration is indicated between brackets; *p < 0.05.

One of the main mechanisms by which the liver regulates the overall cholesterol level is via choleresis. Choleresis is a complex biochemical process leading to the production of bile, an iso-osmotic electrolyte solution that is formed in the liver as a product of its secretory function. It mainly consists of suspended or dissolved organic and inorganic substances in water (Vovkun et al., 2018). Bile is enriched with bile acids (BAs), which are generated from cholesterol processed at the hepatic level. This represents a key step in general cholesterol homeostasis (i.e., excretion pathway) and a potential target for hypercholesterolemia treatment (Forker, 1977; Goldstein and Brown, 1990). The potential ability of the vegetal extracts and their blend, ACFB, to increase the cholesterol-to-bile acid conversion was therefore investigated. As shown in Figure 4 and Supplementary Table S4, the three vegetal extracts significantly increase the production of bile acids in the *in vitro* hepatic model. With about a 17% increase, caigua showed to be the best choleresis-inducing extract. Artichoke and fenugreek extracts increase bile acid production by 9% and 13%, respectively. Atorvastatin increased bile acid production by about 10%, confirming its bile acid synthesis-stimulating activity (Parker et al., 2013; Schonewille et al., 2016). No impact of the different solvents on bile acid biosynthesis was observed (data not shown).

**FIGURE 5:**
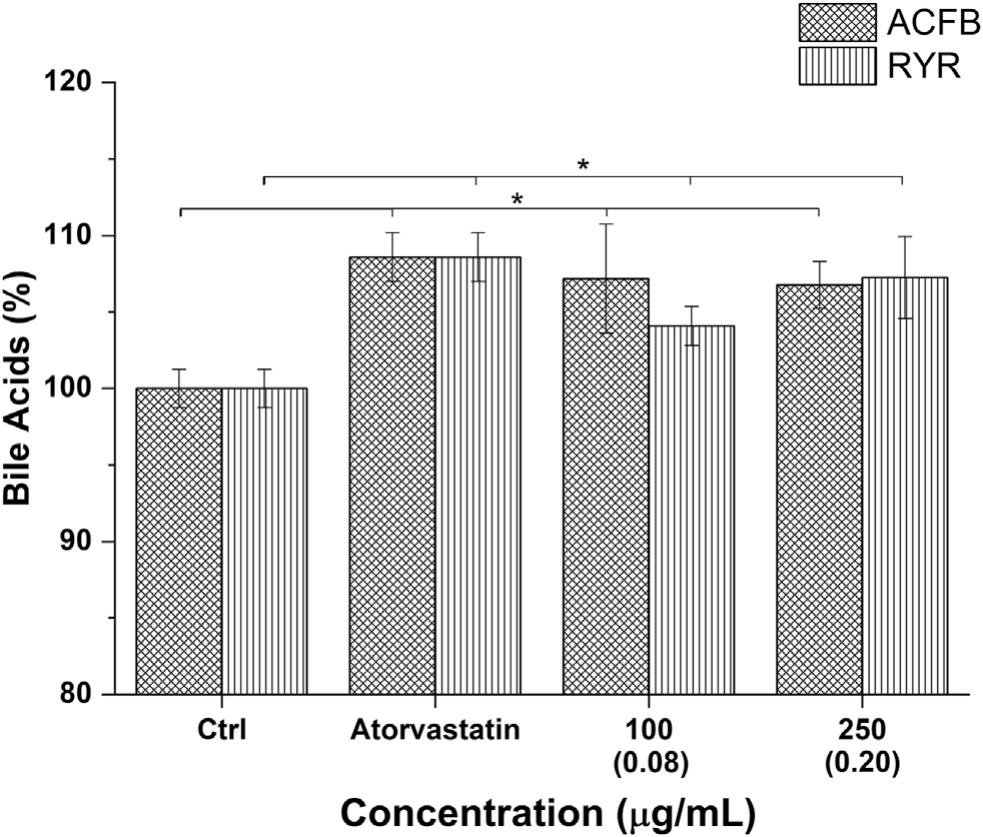
Bile acid production following treatment of hepatic *in vitro* model with atorvastatin (positive control) and different concentrations of ACFB and RIR. Monacolin K concentration is indicated between brackets; *p < 0.05.

The choleretic-stimulating activity of ACFB and RYR was tested at 100 and 250 μg/ml. Both formulations significantly increased bile acid synthesis in the *in vitro* hepatic model in a concentration-independent manner (about 7% for ACFB and 5% for RYR) (Figure 5; Supplementary Table S5). Noteworthy, the increase in bile acids induced by ACFB was achieved with lower vegetal extract concentrations than the single extracts, indicating an additive effect between them. As for the vegetal extracts, no difference in bile acid biosynthesis between different solvents was highlighted (data not shown).

### Impact of vegetal extracts, ACFB, and red yeast rice on hepatic cholesterol biosynthesis

**FIGURE 6:**
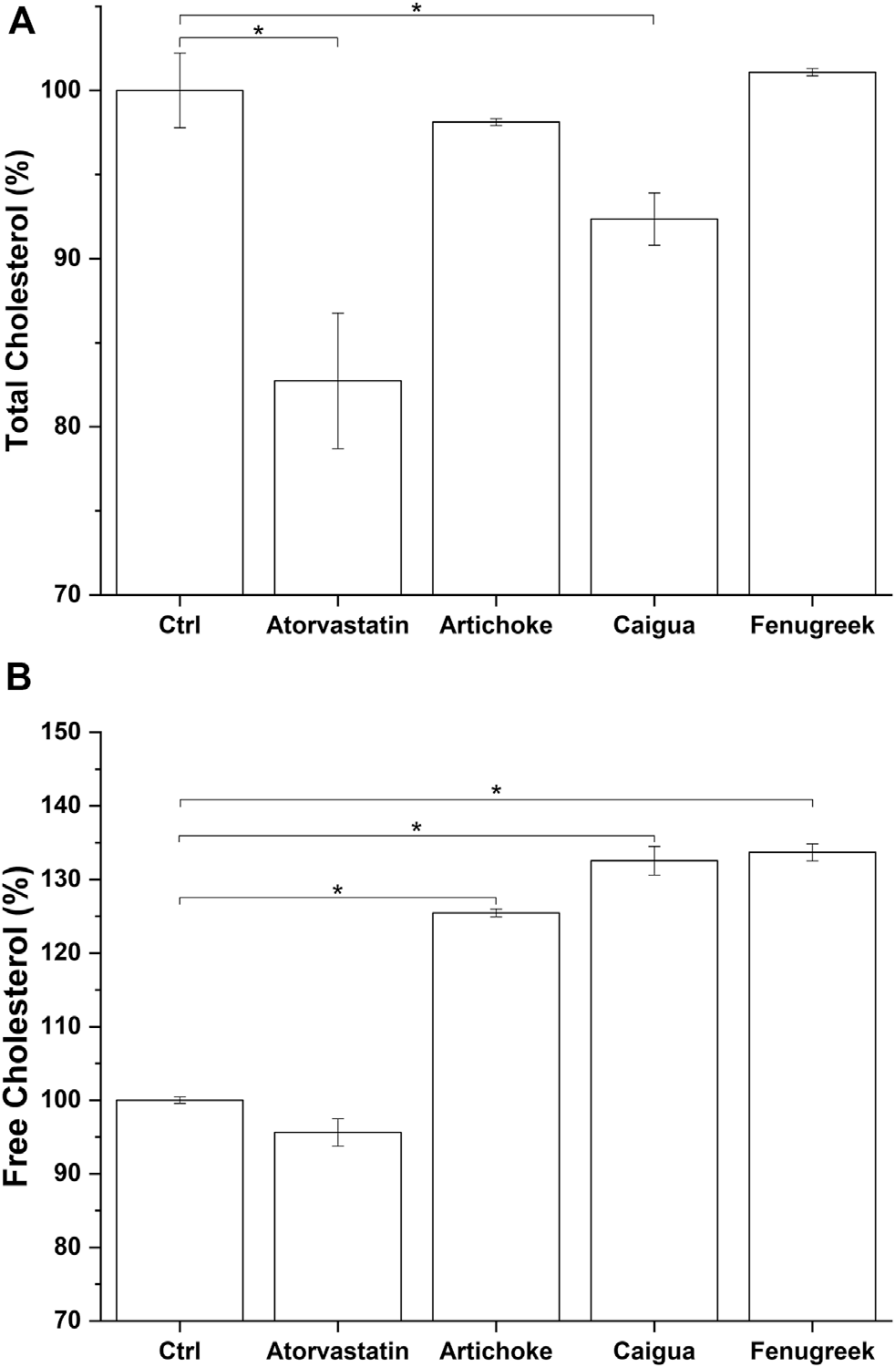
Total cholesterol **(A)** and free cholesterol **(B)** biosynthesis by the hepatic *in vitro* model following 48 h of treatment with atorvastatin and different concentrations of the three vegetal extracts. Monacolin K concentration is indicated between brackets (The result for 100 μg/ml of RICE KOLIN solubilized in ethanol is not shown since it is not statistically different from that at 50 μg/ml). *p < 0.05.

Cholesterol is both synthesized by cells and introduced with food. The liver is the principal site for cholesterol homeostasis maintenance via a plethora of mechanisms, such as biosynthesis, uptake through low-density lipoprotein receptors (LDLr), lipoprotein release in the blood, storage by esterification, and degradation and conversion into bile acids (Weber et al., 2004). Independently from its origin (biosynthesis or food intake), the hepatic cholesterol pool can be enzymatically esterified by AcylCoA-cholesterol acyltransferase and incorporated into very-low-density lipoproteins (VLDL), which are then secreted into the bloodstream for transport to the peripheral tissues (Bays et al., 2008), or excreted as free cholesterol or cholesterol-derived bile acids into the bile and eliminated through the feces (Ikonen, 2006). Consequently, effective treatment for hypercholesterolemia could reduce cholesterol by lowering total cholesterol and/or increasing free cholesterol hepatic content. The latter represents the main form of cholesterol released through bile (Mayes and Botham, 2003; de Boer et al., 2018). Among the tested vegetal extracts, only caigua significantly reduced the total cholesterol content in HepG2 cells (8% reduction) (Figure 6A and Supplementary Table S6), while all the three extracts are effective in increasing free cholesterol production (about 25%, 33%, and 34% for artichoke, caigua, and fenugreek extracts, respectively) (Figure 6B; Supplementary Table S6). As expected, no effect on free cholesterol was observed exposing *in vitro* hepatic model to atorvastatin, while total cholesterol was significantly reduced by about 18% (Figures 6A,B; Supplementary Table S6).

**FIGURE 7:**
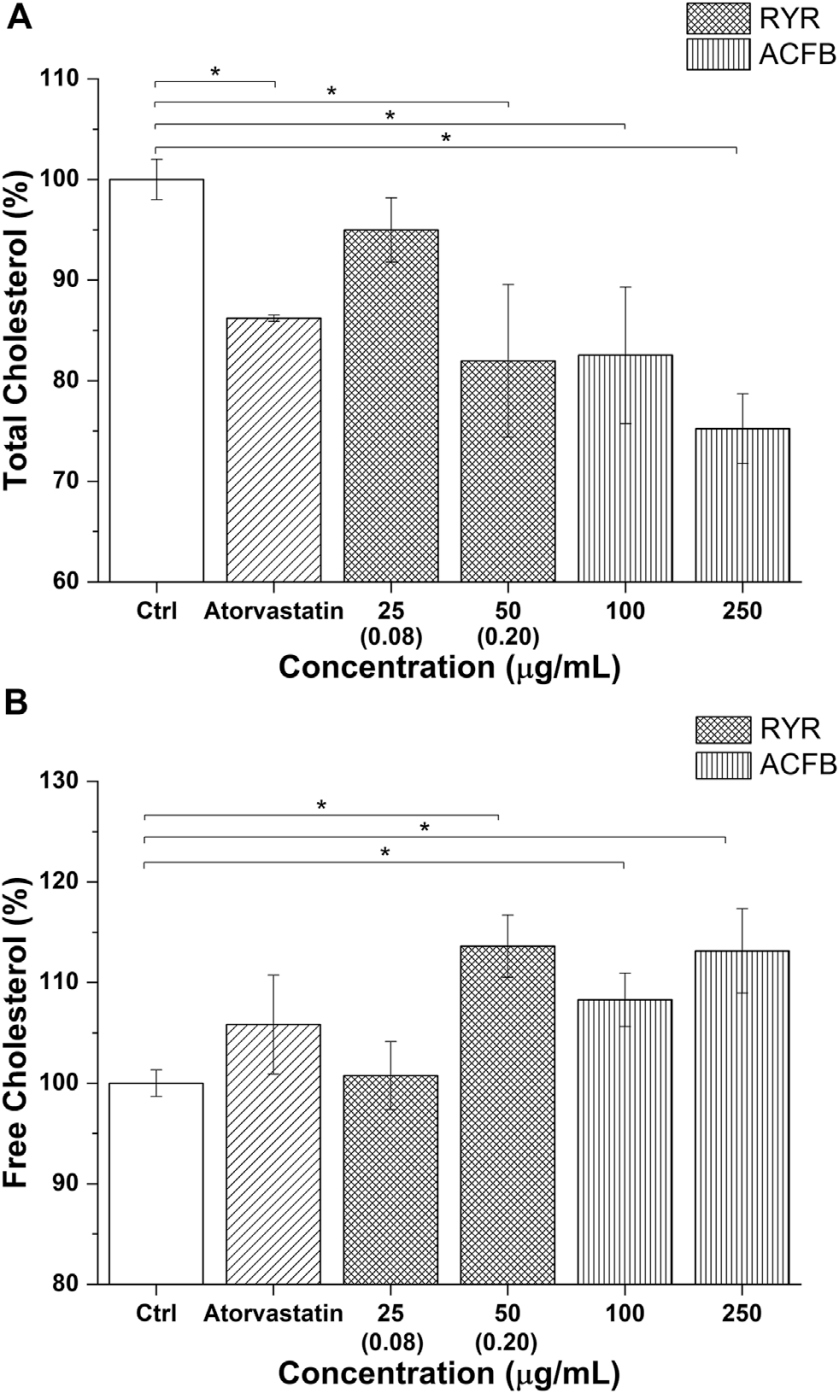
Total cholesterol **(A)** and free cholesterol biosynthesis **(B)** by the hepatic *in vitro* model following 48 h of treatment with atorvastatin and different concentrations of ACFB and RYR formulation. Monacolin K concentration is indicated between brackets (The result for 100 μg/ml of RICE KOLIN solubilized in ethanol is not shown since it is not statistically different from that at 50 μg/ml). *p < 0.05.

The impact on cholesterol metabolism was also tested for ACFB and RYR. Due to poor monacolin K solubilization in DMSO, as confirmed by HPLC analysis (0.08 μg/ml), no effect on cholesterol metabolism was observed (data not shown). Therefore, total and free cholesterol content was measured after treating cells with ethanolic-solubilized rice red extract. Indeed, monacolin K concentration in ethanol is 10 times higher compared with DMSO (Supplementary Table S7). The vegetal extract blend, ACFB, and RYR were tested, respectively, at 100 and 250 μg/ml and 25, 50, and 100 μg/ml. As shown in Figure 7A and Supplementary Table S7, ACFB induces a concentration-independent decrease in total cholesterol (about 20% reduction), while RYR significantly reduces total cholesterol starting from 50 μg/ml, equivalent to a monacolin K concentration of 0.2 μg/ml. No significant effect was observed for RYR at 25 μg/ml. The same behavior was observed for free cholesterol (Figure 7B; Supplementary Table S7). ACFB increases free cholesterol synthesis in HepG2 cells in a concentration-independent way (about 11% increase), while an effect of ethanol-solubilized RYR was highlighted starting from 50 μg/ml. HepG2 cholesterol biosynthesis was not affected following exposure to different solvents (data not shown). As for bile acid synthesis, a possible additive effect between the three vegetal extracts on cholesterol metabolism was highlighted, since significant results were achieved by ACFB with lower vegetal extract concentrations compared with the single vegetal extract.

## Discussion

In the last decades, the research for vegetal extracts endowed with anticholesterolemic activity, but without the statin long-term side effects has catalyzed the efforts of nutraceutical and food supplement industries, in particular, toward novel vegetal extracts used in traditional medicine. Artichoke, caigua, and fenugreek are known to possess antilipidemic and anticholesterolemic effects, and have been used in traditional medicine for the treatment of high lipid and high cholesterol diet-related metabolic diseases (Gonzales et al., 1995; Basch et al., 2003; Shimoda et al., 2003; Vyas et al., 2008; Heidarian et al., 2011; Mohamed Abdel Magied et al., 2016). To the best of our knowledge, this is the first study to investigate anticholesterolemic activity of a novel formulation, ACFB, obtained from the unique blend of these extracts. Our results indicate that ACFB significantly enhances, with a holistic approach, hepatic functionality and cholesterol elimination, by reducing total cholesterol synthesis while improving bile acid and free cholesterol production, as determined by total cholesterol, free cholesterol, and bile acids assays. Indeed, when blended together, the three extracts showed to be more effective than RYR in promoting hepatocyte cholesterol lowering, through a reduction in cholesterol synthesis and its conversion in bile acids (i.e., choleresis promotion). It is also important to underline that such effectiveness in hepatocyte cholesterol lowering was obtained with lower quantities of the extracts, compared with the ones necessary when the extracts were tested individually, indicating a potential additive effect between the extracts themselves. Moreover, to the best of our knowledge, this is the first work to highlight a significant procholeretic activity of caigua, as evidenced by the marked increase in HepG2 bile acid production following exposure to caigua extract. Furthermore, caigua showed to be the extract endowed with the best anticholesterolemic activity, since it shows a significantly higher reduction in total cholesterol and increase in bile acid biosynthesis, while retaining a similar effect on free cholesterol production compared with the other vegetal extracts. Taken together, the experimental findings of the present study primarily confirm the anticholesterolemic efficacy of artichoke, caigua, and fenugreek extract and highlight their increased effectiveness when combined in a unique blend, as in ACFB formulation, suggesting a potential use of this extract to complement or substitute statins. Indeed, while being the main drug for hypercholesterolemia and hyperlipemia treatment, statin-based therapies raised some concerns due to their uncontrolled use and the growing body of evidences highlighting their side effects (Argov, 2015; Pinal-Fernandez et al., 2018; Irvine, 2020). A stark example of statin abuse comes from monacolin K, a natural statin known as lovastatin, extracted from the red yeast *Monascus purpureus* Went. Indeed, despite its efficacy, an alarm was raised by EFSA in the form of a published scientific opinion, since an unharmful dietary intake was not found, for both the general population and vulnerable subgroups of the population (Younes et al., 2018). As such, anticholesterolemic therapies based on novel and safer vegetal extracts and their blends are required to assist or replace statins. Although our results from *in vitro* experiments cannot be directly extrapolated to antilipidemic and anticholesterolemic clinical effects, such studies will assist in screening novel vegetal extracts and blends for their safety and anticholesterolemic efficacy, while starting to elucidate the mechanism through which cholesterol is lowered. To elucidate the molecular targets of these vegetal extracts and their blend, and to investigate other mechanisms responsible for cholesterol homeostasis, more detailed *in vitro* studies will be needed. In parallel to that, an *in vivo* approach will be instrumental in defining the bioavailability of the extracts and their blend and, as a consequence, their anticholesterolemic and antilipidemic efficacy. Finally, DNA barcoding analysis was an efficient tool in uniquely identifying the species of all plants used in this study. DNA testing of raw plants, used as a preliminary analysis, is necessary to select the correct species and, consequently, to obtain the suitable phytocomplexes for the ACFB. To the best of our knowledge, it was demonstrated, for the first time that caigua was also grown in Val Camonica (Lombardy, Italy) under the vernacular name of “milione” or “miliun.” This finding could positively impact on the local agriculture in mountain areas and may lead to a more sustainable supply chain for its use in food and food supplements.

In conclusion, our results suggest that combining active ingredients coming from traditional herbal medicine, such as artichoke, caigua and fenugreek, in a unique blend like ACFB, could enhance their curative effects, potentially providing complementary therapies to the statin-based ones for hyperlipidemia and hypercholesterolemia-related complications.

## Data Availability

The raw data supporting the conclusion of this article will be made available by the authors, without undue reservation.
