# Supplementary material for: Anticholesterolemic Activity of Three Vegetal Extracts (Artichoke, Caigua, and Fenugreek) and Their Unique Blend
Source: Front Pharmacol. 2021 Nov 23;12:726199. doi: 10.3389/fphar.2021.726199 (PMC8650624; doi:10.3389/fphar.2021.726199)
Supplement: Supplementary file 1 [file DataSheet2.PDF]

Supplementary Material

1 Supplementary Figures

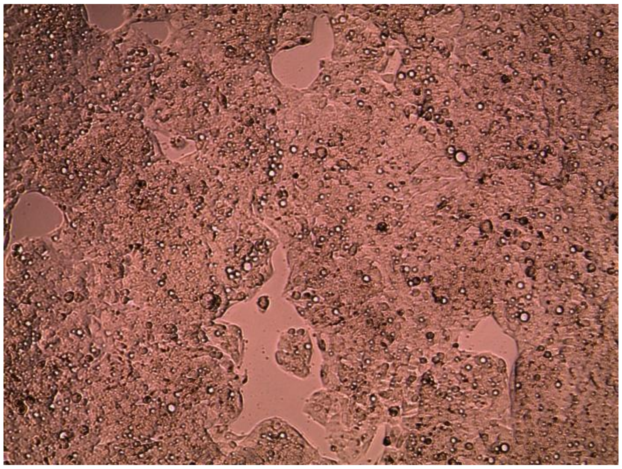

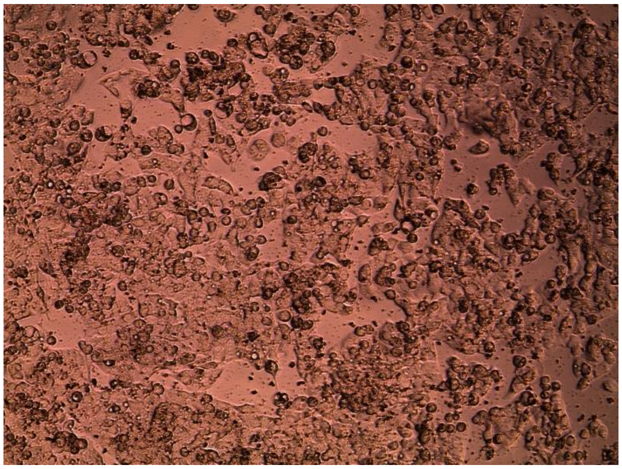

Ctrl **750 µg/mL**

Supplementary Figure 1. Morphological impact of treatment with 750 µg/mL of OMEOLIPID formulation on the hepatic in vitro model.

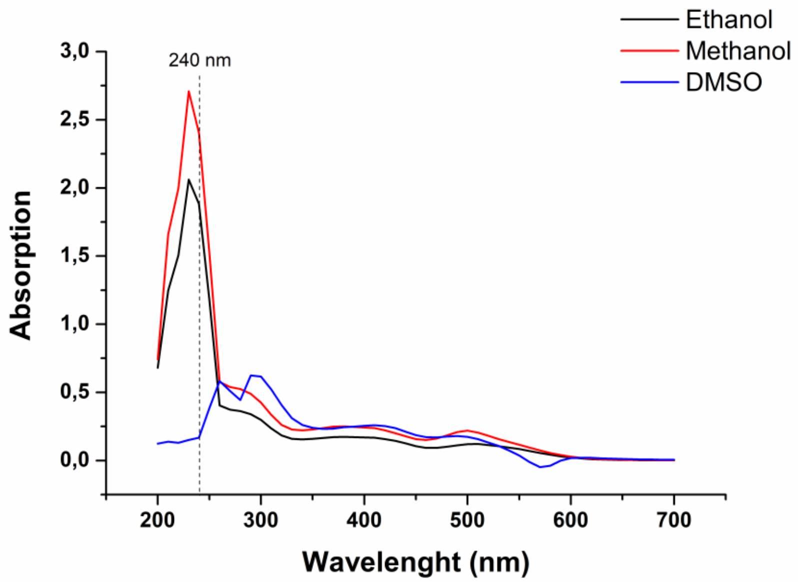

Supplementary Figure 2. The absorption spectrum of *RYR* following solubilization with ethanol (black line), methanol (red line) and DMSO (blue line). The dashed line highlights the absorption peak for monacolin K (240 nm). Both alcoholic solvents (i.e. ethanol and methanol) significantly improved monacolin K solubilization from RYR, compared to DMSO. In particular, ethanol increases monacolin

K solubilization by 10 times, with a final concentration of 0.80 µg/mL in the supernatant, slightly lower compared to methanol solubilization (12 fold increase; final monacolin K concentration 0.96 µg/mL).

2 Supplementary Tables

Supplementary Table 1. List of tested extracts and formulations and their commercial name.

Extract/Formulation	Commercial Name
Cynara scolymus extract	Artichoke
Cyclanthera pedata extract	Caigua
Trigonella foenum-graecum extract	Fenugreek
Cynara scolymus, Cyclanthera pedate, Trigonella foenum-graecum blend (ACFB)	OMEOLIPID<br>(Artichoke 40-60 %; Caigua 20-40 %; Fenugreek 20-40%)
Monascus purpureus extract	Red Yeasy Rice

Supplementary Table 2. In the table are shown primer name, primer sequence (5'-3'), region marker and the reference.

**Primer name**	**5'-3'**	**Region marker**	**Reference**
rbcL_1F	ATGTCACCACAAACAGAAAC	*rbcL*	36
rbcL_724R	TCGCATGTACCTGCAGTAGC		
psbA	GTTATGCATGAACGTAATGCTC	*psbA-trnH*	37
trnH	CGCGCATGGTGGATTCACAATCC		

Supplementary Table 3. In the table are shown sample ID, declared and verified species after DNA barcoding analysis

**Sample ID**	**Declared and verified species**
DB574_A	*Cyclanthera pedata*
DB574_B	*Cyclanthera pedata*
DB574_C	*Cyclanthera pedata*
DB574_D	*Cyclanthera pedata*
DB574_E	*Cyclanthera pedata*
DB690_A	*Cyclanthera pedata*
DB690_B	*Cyclanthera pedata*
DB690_C	*Cyclanthera pedata*

DB690_D	*Cyclanthera pedata*
DB690_E	*Cyclanthera pedata*
DB304_A	*Cynara scolymus*
DB304_B	*Cynara scolymus*
DB304_C	*Cynara scolymus*
DB304_D	*Cynara scolymus*
DB304_E	*Cynara scolymus*
DB333_A	*Trigonella foenum-graecum*
DB333_B	*Trigonella foenum-graecum*
DB333_C	*Trigonella foenum-graecum*
DB333_D	*Trigonella foenum-graecum*
DB333_E	*Trigonella foenum-graecum*

Supplementary Table 4. Bile acids obtained following treatment of the hepatic in vitro model with atorvastatin and the three vegetal extracts. Results are expressed as a percentage compared to control (mean ± standard deviation).

**Treatment**	**Bile acids (%)**
Atorvastatin	111.4 ± 5.4
Artichoke	108.8 ± 0.3
Caigua	117.0 ± 1.0
Fenugreek	113.4 ± 0.8

Supplementary Table 5. Bile salts obtained following treatment of the hepatic in vitro model with atorvastatin, OMEOLIPID and RYR. Results are expressed as a percentage compared to control (mean ± standard deviation).

Treatment	Bile Acids (%)
Ctrl	100.0 ± 1.3
Atorvastatin (5 µg/mL)	108.6 ± 1.6
OMEOLIPID (100 µg/mL)	107.2 ± 3.6
OMEOLIPID (250 µg/mL)	106.8 ± 1.5
RYR (100 µg/mL)	104.1 ± 1.3
RYR (250 µg/mL)	107.2 ± 2.7

Supplementary Table 6. Total and free cholesterol biosynthesis obtained following treatment of the hepatic in vitro model with atorvastatin and artichoke, caigua and fenugreek extracts. Results are expressed as a percentage compared to control (mean ± standard deviation).

Treatment	Total Cholesterol (%)	Free cholesterol (%)
Atorvastatin	82.7 ± 2.2	95.6 ± 1.8
Artichoke	98.1 ± 0.2	125.5 ± 0.5
Caigua	92.3 ± 1.6	132.6 ± 1.9
Fenugreek	101.1 ± 0.2	133.7 ± 1.2

Supplementary Table 7. Total and free cholesterol biosynthesis obtained following treatment of the hepatic in vitro model with atorvastatin, OMEOLIPID and RYR solubilized with ethanol at different concentration. Results are expressed as a percentage compared to control (average ± standard deviation).

**Treatment**	**Total Cholesterol (%)**	**Free Cholesterol (%)**
Ctrl	100.0 ± 2.0	100.0 ± 1.3
Atorvastatin (5 µg/mL)	86.2 ± 0.3	105.8 ±4.9
RYR (25 µg/mL)	95.0 ± 3.2	100.8 ± 3.4
RYR (50 µg/mL)	81.9 ± 7.6	113.6 ± 3.1
RYR (100 µg/mL)	77.6 ± 1.4	120.5 ± 2.1
OMEOLIPID (100 µg/mL)	82.5 ± 6.8	108.3 ± 2.6
OMEOLIPID (250 µg/mL)	75.2 ± 3.5	113.2 ± 4.2
