# Supplementary figures and images for: Anticholesterolemic Activity of Three Vegetal Extracts (Artichoke, Caigua, and Fenugreek) and Their Unique Blend
Source: Front Pharmacol. 2021 Nov 23;12:726199. doi: 10.3389/fphar.2021.726199 (PMC8650624; doi:10.3389/fphar.2021.726199)

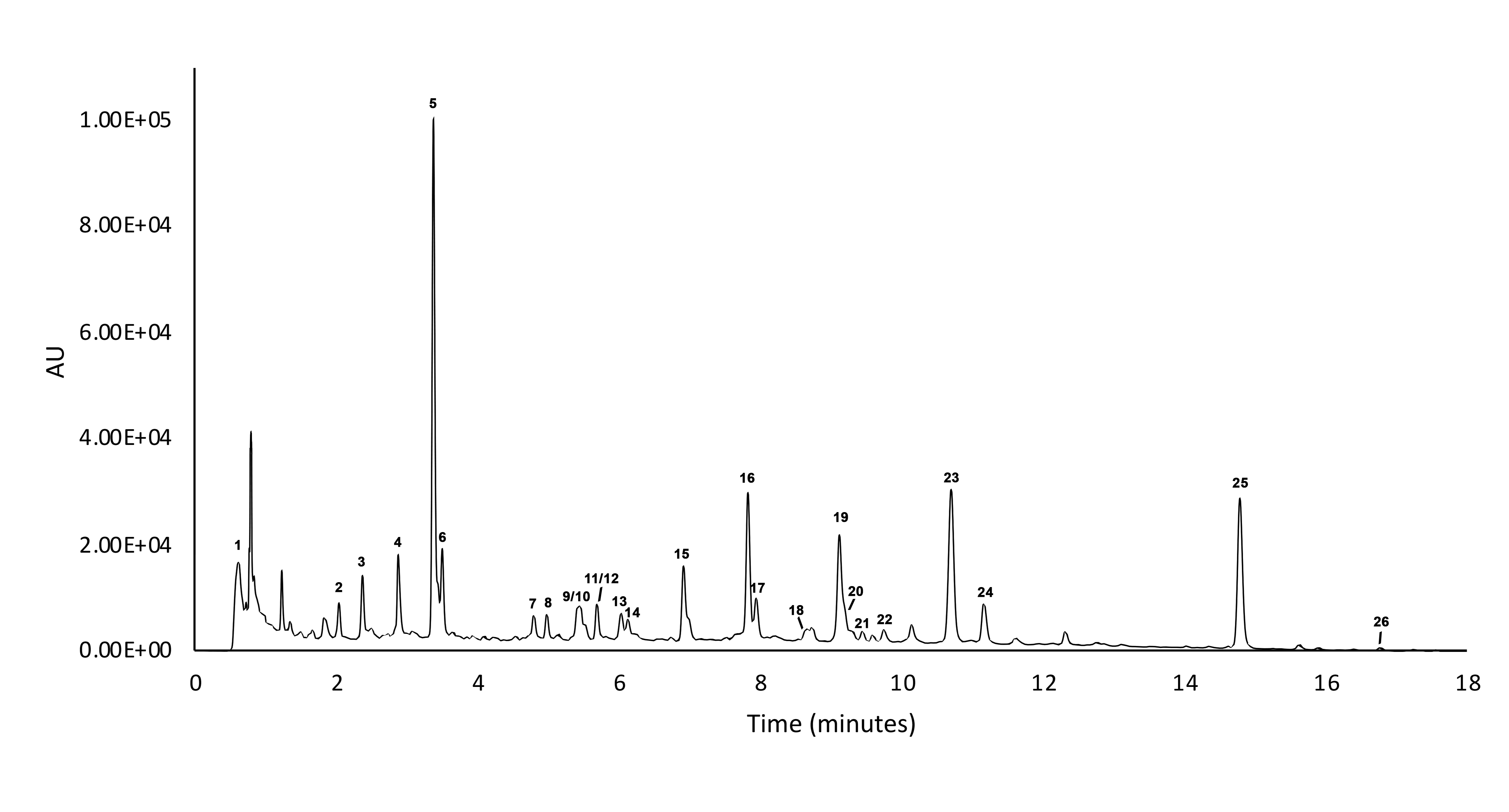

Supplement: Supplementary file 2 [file DataSheet1.ZIP › Figure 1.jpg]
